# Evaluation of the Tobbstop Mobile App for Smoking Cessation: Cluster Randomized Controlled Clinical Trial

**DOI:** 10.2196/15951

**Published:** 2020-06-26

**Authors:** Meritxell Pallejà-Millán, Cristina Rey-Reñones, Maria Luisa Barrera Uriarte, Esther Granado-Font, Josep Basora, Gemma Flores-Mateo, Jordi Duch

**Affiliations:** 1 Unitat de Suport a la Recerca Camp de Tarragona Fundació Institut Universitari per a la recerca a l'Atenció Primària de Salut Jordi Gol i Gurina Reus Spain; 2 Departament de Ciències Mèdiques Bàsiques Universitat Rovira i Virgili Reus Spain; 3 Institut Català de la Salut Unitat de Suport a la Recerca Camp de Tarragona Reus Spain; 4 Equip d'Atenció Primaria La Granja (Tarragona-2) Direcció d'Atenció Primaria Camp de Tarragona Institut Català de la Salut Torreforta Spain; 5 Equip d'Atenció Primaria Horts de Miró (Reus-4) Direcció d'Atenció Primaria Camp de Tarragona Institut Català de la Salut Reus Spain; 6 Unitat d’Anàlisi i Qualitat Xarxa Sanitària i Social Santa Tecla Tarragona Spain; 7 Departament d’Enginyeria Informàtica i Matemàtiques Universitat Rovira i Virgili Tarragona Spain

**Keywords:** tobacco smoking, tobacco use cessation, mobile application, primary public health, clinical trial, mobile phone

## Abstract

**Background:**

Mobile apps provide an accessible way to test new health-related methodologies. Tobacco is still the primary preventable cause of death in industrialized countries, constituting an important public health issue. New technologies provide novel opportunities that are effective in the cessation of smoking tobacco.

**Objective:**

This paper aims to evaluate the efficacy and usage of a mobile app for assisting adult smokers to quit smoking.

**Methods:**

We conducted a cluster randomized clinical trial. We included smokers older than 18 years who were motivated to stop smoking and used a mobile phone compatible with our mobile app. We carried out follow-up visits at 15, 30, and 45 days, and at 2, 3, 6, and 12 months. Participants of the intervention group had access to the Tobbstop mobile app designed by the research team. The primary outcomes were continuous smoking abstinence at 3 and 12 months.

**Results:**

A total of 773 participants were included in the trial, of which 602 (77.9%) began the study on their D-Day. Of participants in the intervention group, 34.15% (97/284) did not use the app. The continuous abstention level was significantly larger in the intervention group participants who used the app than in those who did not use the app at both 3 months (72/187, 38.5% vs 13/97, 13.4%; *P*<.001) and 12 months (39/187, 20.9% vs 8/97, 8.25%; *P*=.01). Participants in the intervention group who used the app regularly and correctly had a higher probability of not being smokers at 12 months (OR 7.20, 95% CI 2.14-24.20; *P*=.001) than the participants of the CG.

**Conclusions:**

Regular use of an app for smoking cessation is effective in comparison with standard clinical practice.

**Trial Registration:**

Clinicaltrials.gov NCT01734421; https://clinicaltrials.gov/ct2/show/NCT01734421

## Introduction

The number of health interventions involving mobile-based technologies has been increasing in recent years, given the potential of reaching larger audiences who own and use smartphones on a daily basis. In the case of Spain, between 80% and 90% of the population have at least one smartphone [1], which places the country at the top of European mobile phone usage, with 23 million people owning smartphones [2]. Mobile apps provide an accessible way to test new health-related methodologies, which also address user concerns around the availability and confidentiality of their personal data [3]. In Spain, users download around 4 million apps every day and, more importantly, two-thirds of teenagers (and young users) downloaded and used a mobile health app in the last year.

Tobacco consumption levels around the world vary significantly; while in some low-income countries there has been an increase in the prevalence of tobacco, in industrialized countries the observed decrease in consumption in recent years seems to have stopped. Nonetheless, tobacco is still the primary preventable cause of death in industrialized countries, constituting an important public health issue despite all the medical advances and resources invested to reduce tobacco-related death and diseases [4-7].

There are many available mobile apps designed to support smokers in the process of tobacco cessation. However, most of these apps lack scientific evidence that prove their effectiveness [8]. In a recent systematic review [9] focused on analyzing available apps that support the process of smoking cessation, the authors found that only 6 out of 158 apps are supported by low-quality scientific evidence, 3 of which are currently available on smartphone markets, and only 2 of which are in the top 50 most popular apps for smoking cessation [9]. This creates two important issues: the limited number of scientifically validated apps and the unavailability of such apps for the general population. In order to increase the quality and the availability of apps, experts suggest that it is necessary to develop an innovative framework capable of scientifically evaluating different properties of the apps, and then improve distribution channels to make those certified apps easier to find for the consumer. Moreover, certified apps will also be easier to use in health care environments, given that they should comply with a set of standards and regulations [10]. Official institutions also suggest the use of simple language to widen the dissemination of health-related information.

Our research team has been working since 2013 to evaluate the efficacy of a gamified mobile app with the goal of increasing the success rate of smoking cessation interventions in adults (individuals older than 18 years) that are already motivated to quit smoking.

## Methods

### Study Design

The protocol of the study was previously published [11] and was executed in 2 phases. First, an interdisciplinary team composed of doctors, nurses, educators, designers, and computer engineers designed and implemented the Tobbstop mobile app, which combines gamification principles with the latest mobile technologies to create a novel experience designed to follow a tobacco withdrawal guide. Second, a cluster randomized clinical trial was conducted in order to evaluate the efficacy of the mobile app and the features included within.

### Setting

We included participants from the primary health care regions of Tarragona and Terres de l’Ebre (Catalonia, Spain).

### Recruitment of Primary Health Care Professionals

The participation of the primary health care professionals was voluntary. Health professionals—doctors and nurses—were informed of the goals and scope of the study, provided with the study protocol [11] and related documentation, and trained by the members of the research team to inform them of the details of the study. Individuals that participated in the study were recruited by the health professionals, provided that they met our inclusion criteria.

### Participant Eligibility Criteria

Our inclusion criteria were (1) current smokers aged 18 years or older who smoked at least 10 cigarettes per day, (2) owners of an Android or iOS (Apple Inc) mobile phone compatible with our mobile app, and (c) smokers with a moderate-high motivation to quit smoking (Richmond test score ≥5) [12].

The exclusion criteria were (1) patients addicted to psychoactive substances other than tobacco, (2) patients with a psychotic disorder, (3) patients without a smartphone with the minimum hardware requirements necessary to run the app, and (4) participants who had a low motivation to quit smoking (Richmond test score <5).

If a participant did not meet the motivation criterion, health professionals provided information and measures to increase motivation and arranged a second session to try to recruit the participant into our study.

### Random Allocation

The unit of randomization was the primary health care centers that participated in the recruiting. Each center enrolled was assigned randomly to one of the 2 groups (control or intervention). Centers were stratified according to rural or urban locations and the number of health professionals available in each of them. We ensured comparability between the 2 groups. For the randomization, we used the software EpiData (version 3.0, EpiData Association).

### Blinding

Given the nature of the intervention we could not prevent the participants and health professionals from knowing to which of the 2 groups they were assigned. However, in the data analysis phase, we blinded the data so no identification process could be carried out.

### Intervention

During the recruitment process (June 2014-May 2016) professionals invited active smokers older than 18 years. The participants that met the different selection criteria and were willing to participate were asked to sign a written consent form.

In the first visit (defined as visit 0), we collected demographic data, asked the participant to fill in the study questionnaire, and fixed the D-Day upon which they would initiate the process of smoking cessation. A few days before the D-Day, each participant had a second visit, where the smoker and the health professional discussed the plan to be followed throughout the process, in accordance with the Clinical Practice Guidelines of the Institut Català de la Salut—Catalan Health Institute [13]—and planned all the remaining monitoring visits.

Both groups received information on the standard guidelines of clinical practice. In the event that a participant did not attend a follow-up meeting, professionals called their phone to reintroduce them into the study. If they could not be reached, they were considered as relapsed as of that point.

### Description of the Mobile App

The participants assigned to the intervention group (IG) received a numeric code to activate their access to the Tobbstop app and a detailed explanation of its basic features. This app is included in the *Serious Games* category, which are apps that include components from games designed to facilitate the achievement of the goals of the app, increasing user engagement and improving user experience. Tobbstop was designed with the goal of engaging the participants to use the app for at least the 3-month period that they were in the clinical study, and it included features to motivate them to use the app every day during this period. We also included features that covered Bartle’s taxonomy of player types (killers, socializers, achievers, and explorers) and that adapted to the different stages of the tobacco withdrawal process: start, euphoria, grief, normalization, and consolidation. All this work was performed with an interdisciplinary team of experts that included experts on tobacco withdrawal (in smokers and ex-smokers), computer engineers, graphic designers, game designers, educators, and health professionals (doctors and nurses). For more details about the app features, see Multimedia Appendix 1.

The clinical trial was designed to measure success 12 months after the start of the study, but the app was only designed to cover the first 3 months. Once participants completed the path through the island in the app, they could continue using the app and all the features provided without limitation.

### Control Group

Participants assigned to the control group (CG) only followed the recommendations of the health professionals included in the study [13].

### Data Collection

During the first visit, we collected all sociodemographic and anthropometric data, such as date of birth, gender, level of education, civil status, weight (kg), height (cm), and blood pressure (mmHg), as well as data on the presence of other pathologies, such as high blood pressure, chronic obstructive pulmonary disease, diabetes mellitus, stroke, neoplasia, acute myocardial infarction, dyslipidemia, angina pectoris, and intermittent claudication. We also collected data on tobacco consumption, including number of cigarettes smoked per day, usage of electronic cigarettes, age at which the individual started smoking, number of previous attempts to quit smoking, longest period without smoking, and presence of other smokers in the family. Nicotine dependence level was measured using the Fagerström test [14] and their motivation was measured using the Richmond test [12].

In each of the follow-up visits we measured weight and blood pressure and asked about tobacco consumption and the existence of abstinence syndrome. Tobacco abstinence was confirmed by the level of carbon monoxide (CO) in exhaled breath. We recorded any other treatments used for tobacco cessation (ie, pharmacological treatment).

If participants did not attend the follow-up interviews, we attempted to call them by phone. In some cases, when the participant was unable to attend the visit at the health center, we conducted the follow-up interview via phone in order to ensure the participant’s continuance with the study, but in such cases we were unable to collect all the measures (weight, height, CO-oximetry).

The follow-up period had the following end-points: (1) when the participant did not attend one of the visits and we were unable to contact them thereafter, (2) when the participant decided to quit the study, (3) when the participant started to smoke again, or (4) after 12 months without smoking.

### Computed Variables

We computed the body mass index using height and weight and mean arterial pressure according to the formula [(diastolic arterial pressure x 2) + systolic arterial pressure] / 3.

### Sample Size

To compute the sample size, which was randomized by the primary health care centers that participated, we multiplied the number of individuals required by the design effect. We accepted an α risk of 5% and a β risk of 20% in a bilateral contrast. We counted 222 participants in each group, detecting a difference of less than 5%, with measurements based on Epidat (version 3.1; Xunta de Galicia). In order to compute the design effect, we estimated an intracluster correlation coefficient in randomized clinical trials, which is usually lower than 0.05. The average size was 20 with a design effect of 1.36. Using all these values, we set the sample size to 604 participants, with 302 in each group.

### Statistical Analysis

We performed intention-to-treat analysis to evaluate the comparability of the 2 groups.

Quantitative measures are described with mean and standard deviation if they have a normal distribution, or median and interquartile range if they do not. Qualitative variables are described with percentages and 95% confidence intervals. Baseline quantitative measures are compared using Student’s *t* test, while qualitative measures are compared using Pearson’s chi-square test.

The primary outcomes were continuous abstinence at 3 and 12 months.

We evaluated the app efficacy using a protocol analysis, comparing the CG participants with the IG participants who used the app. We computed the crude and adjusted hazard ratios using a multilevel Cox regression with 2 effects (fixed and random). The fixed component included group assignment and, in the adjusted models, sociodemographic variables. The random component included assignment to a primary health care center. Data analysis was performed using R version 3.4.3 [15].

### Ethical Aspects of the Study

The study, in its revised and updated version, was carried out following the Declaration of Helsinki principles and the Spanish Clinical Practice Guidelines. The study protocol was approved by the Clinical Research Ethics Committee at the Institut Universitari d'Investigació en Atenció Primària Jordi Gol. Data confidentiality was guaranteed by the Spanish law that regulated the protection of personal data at the time of the study, the Ley Orgánica de Protección de Datos de Carácter Personal (15/1999, December 13).

## Results

### Participant Characteristics

**Figure 1:**
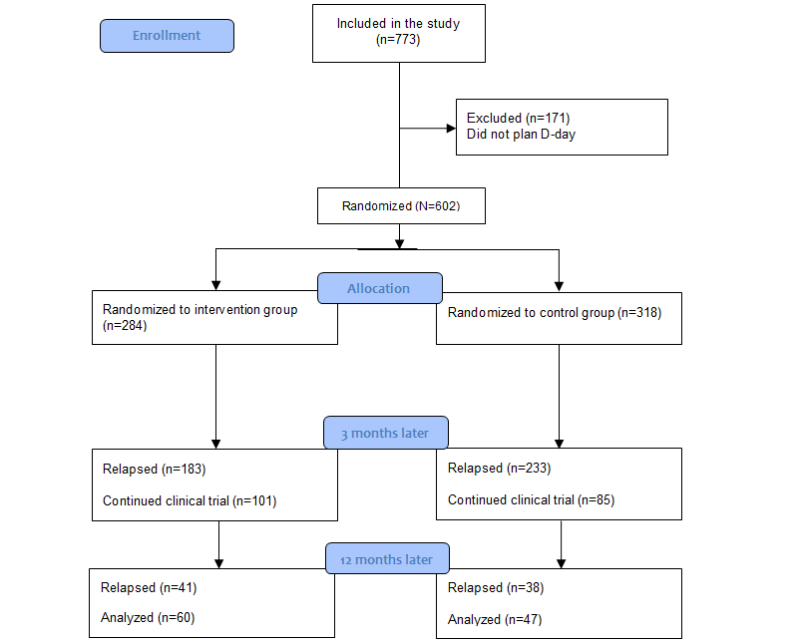
Flowchart of the participants included in the analysis.

We recruited 773 participants for the study, of which 602 (77.9%) started the study on their D-Day. In Figure 1 we detail the flow of the participants within the study [16].

The participants who set their D-Day belonged to one of the 22 health care centers assigned to the IG, and they were treated by 67 health professionals (nurses and doctors). There were no significant differences in the basic characteristics of the participants prior to the intervention if we compare the centers in which they were recruited or the professionals who recruited them. The basic characteristics of the participants are detailed in Table 1. Even though there were not large differences between the 2 groups, we observed that the IG participants were younger than those in the CG (42.2 years vs 48.8 years; *P*<.001), and they had a higher education level (*P*=.001), lived alone (*P*=.03), had a lower CO level (*P*=.001), had lower dependence (*P*=.02), had more family members who smoke (*P*=.03), and had lower blood pressure (*P*=.003) than participants in the CG. The CG used more pharmacological treatment for tobacco withdrawal (*P*<.001).

When looking at the efficacy of the study by participant group, we observed that there were no significant statistical differences between the 2 groups, with slightly better results observed in the CG.

**Table 1:** Basic characteristics of the Tobbstop study participants based on their assigned group.

Characteristics			Control group (n=318)		Intervention group (n=284)		*P* value		Total (n=602)
Mean age, years (SD)			48.8 (11.0)		42.2 (10.2)		<.001		45.7 (11.1)
Men (%)			155 (48.7)		120 (42.3)		.13		275 (45.7)
**Education, n (%)**							.001		
	No studies or primary	126 (39.6)		70 (24.7)				196 (32.6)	
	Secondary	129 (40.6)		143 (50.5)				272 (45.3)	
	University or higher	63 (19.8)		70 (24.7)				133 (22.1)	
**Marital status, n (%)**							.03		
	Single	55 (17.3)		72 (25.4)				127 (21.1)	
	With a partner	205 (64.5)		171 (60.2)				376 (62.5)	
	Widowed	15 (4.7)		5 (1.8)				20 (3.3)	
	Divorced	43 (13.5)		36 (12.7)				79 (13.1)	
Body mass index^a^ (kg/m^2^), mean (SD)			27.4 (8.9)		26.9 (8.5)		.47		27.2 (8.7)
Mean arterial pressure^a^ (mm/Hg), mean (SD)			91.4 (10.6)		60.5 (10.7)		.30		91 (10.7)
CO^b^-oximetry (ppm), mean (IQR^c^)			16.8 (8.00-21.00)		13.6 (6.00-20.00)		.001		15.2 (6.75-20.00)
Electronic cigarette users, n (%)			49 (15.4)		54 (19)		.29		103 (17.1)
**Cigarettes per day, n (%)**							.12		
	0-10	60 (18.9)		73 (25.7)				133 (22.1)	
	11-20	183 (57.5)		154 (54.2)				337 (56.0)	
	21-30	60 (18.9)		38 (13.4)				98 (16.3)	
	31-40	12 (3.7)		15 (5.3)				27 (4.5)	
	>40	3 (0.9)		4 (1.4)				7 (1.2)	
**Fagerström test (dependence), n (%)**							.02		
	High	37 (11.6)		35 (12.3)				72 (12.0)	
	Moderate	217 (68.2)		165 (58.1)				382 (63.5)	
	Low	64 (20.1)		84 (29.6)				148 (24.6)	
Number of previous attempts, mean (IQR)			1.90 (1.00-3.00)		2.61 (1.00-3.00)		.08		2.24 (1.00-3.00)
Maximum number of withdrawal months, mean (SD)			13.8 (31.1)		10.4 (20.9)		.12		12.2 (26.8)
Age of starting smoking (years), mean (SD)			17.0 (4.1)		16.7 (3.4)		.33		16.8 (3.8)
No smokers in the family, n (%)			154 (48.4)		111 (39.1)		.03		265 (44.0)
Use of a pharmacological treatment for tobacco cessation, n (%)			244 (76.7)		148 (52.1)		<.001		392 (65.1)
**Pathologies, n (%)**									
	Hypertension	72 (22.6)		37 (13.0)		.003		109 (18.1)	
	Chronic obstructive pulmonary disease	24 (7.5)		11 (3.9)		.08		35 (5.8)	
	Type 2 diabetes mellitus	23 (7.2)		16 (5.6)		.53		39 (6.5)	
	Stroke	3 (0.9)		5 (1.8)		.49		8 (1.3)	
	Neoplasia	5 (1.6)		6 (2.11)		.85		11 (1.8)	
	Dyslipidemia	83 (26.1)		55 (19.4)		.06		138 (22.9)	
	Coronary heart disease	16 (5)		6 (2.1)		.09		22 (3.6)	
3-month withdrawal, n (%)			101 (31.8)		85 (29.9)		.69		186 (30.9)
12-month withdrawal, n (%)			60 (18.9)		47 (16.5)		.53		107 (17.8)

^a^At baseline.

^b^CO: carbon monoxide.

^c^IQR: interquartile range.

### App Usage

Out of the 284 participants enrolled in the IG, 97 (34.1%) did not use the app, never used the code to activate the app, or did not send any data on their usage from their mobile phone to our server.

We observed significant statistical differences between the participants who used the app and those who did not. Those who did not use the app had lower CO levels (*P*=.03) and smoked more cigarettes per day (*P*=.04). The IG participants who did not use the app indicated less pharmacological treatment than those who used the app (41/97, 42.3% vs 107/187, 57.2%; *P*=.02).

**Figure 2:**
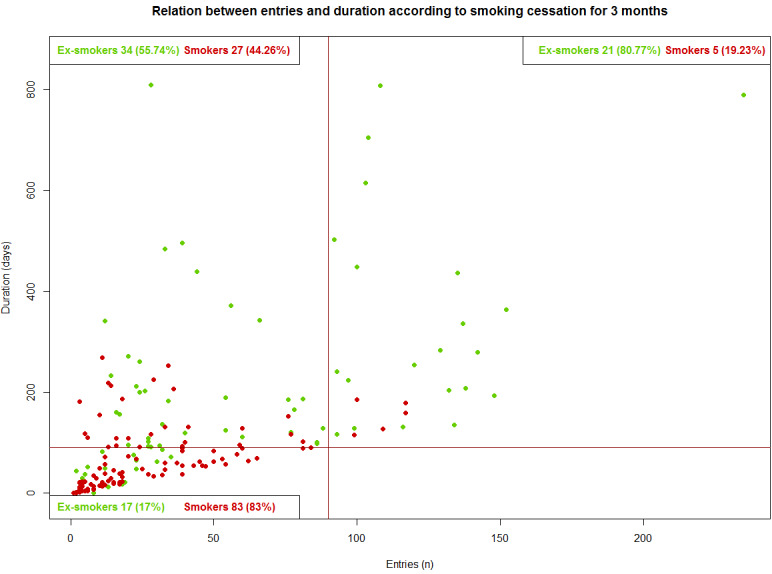
Relation between entries and duration according to smoking cessation for 3 months.

**Figure 3:**
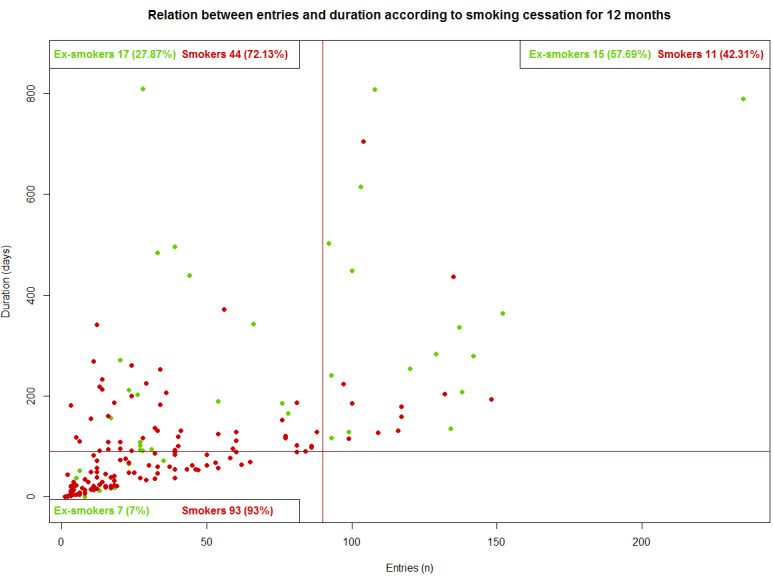
Relation between entries and duration accoding to smoking cessation for 12 months.

Finally, we observed that abstinence was significantly larger in the IG participants who used the app than in those who did not use the app at both 3 months (72/187, 38.5% vs 13/97, 13.4%; *P*<.001) and 12 months (39/187, 20.9% vs 8/97, 8.2%; *P*=.01; Figures 2 and 3).

### Results According to the App Features Used

**Figure 4:**
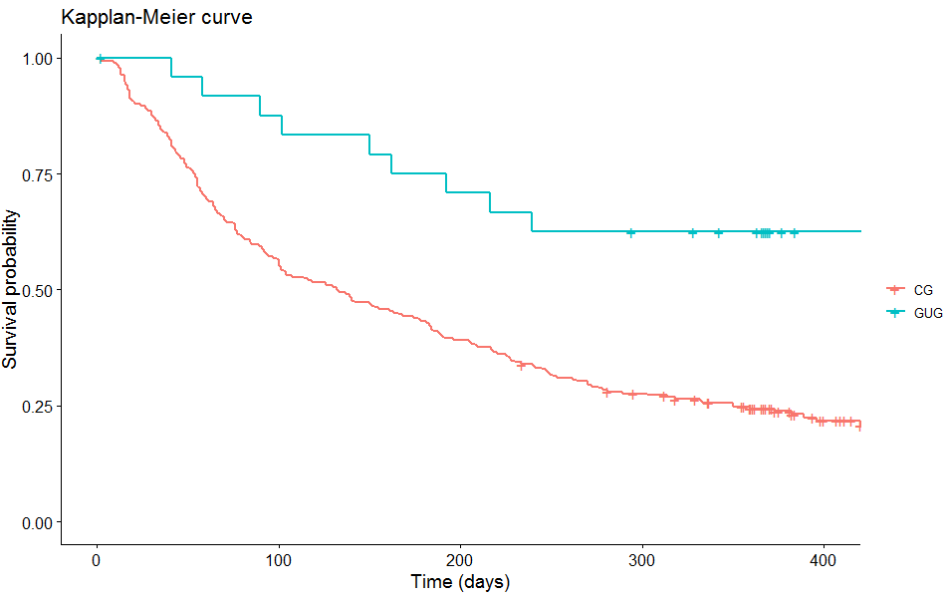
Kapplan-Meier curve GC (control group) vs GUG (good users group).

We consider the good users of the app (based on our design goals) to be those who used the app for at least 90 days and accessed the app at least 90 times. In accordance with these 2 conditions, we consider 26 of the 187 participants who used the app at least once to be good users. Among the main differences, we observed that the participants of the good users group smoked fewer cigarettes per day, had smaller dependencies according to the Fagerström test, and used less pharmacological treatment than those in the CG. The 3-month abstinence of the participants included in the good users group was 80.8% (21/26) compared with 31.7% (51/161) in the other users (*P*<.001). The 12-month abstinence rate for the good users was 57.7% (15/26) compared with 14.9% (24/161) for the other users of the app (*P*<.001; Figure 4).

We evaluated which app features were used the most by the IG. Those who succeeded were more active users of the app and logged in more than twice as much as the other participants. With the exception of the chat feature, which was used independently of whether the participant relapsed, those who did not relapse used the app features more than those who relapsed, as seen in Table 2. Out of the 187 participants who used the app, 38.5% (15/39) of the ex-smokers were considered good users, while only 7.4% (11/148) of those who relapsed were good users (*P*<.001).

**Table 2:** List of a set of app metrics, classified according to the success rate at the end of the study (12 months from the day at which they stopped smoking) for participants of the intervention group who used the app (n=187).

Characteristics			Relapse (n=148)		Abstinence at 12 months (n=39)		*P* value
Pharmacological treatment, n (%)			91 (61.5)		16 (41.0)		.03
**App metrics**							
	Duration within the app (days), mean (SD)	85.2 (94.8)		262 (220.0)		<.001	
	Number of different days connected, mean (SD)	31.7 (33.0)		67.4 (53.5)		<.001	
	Chat usage, n (%)	78 (52.7)		23 (59.0)		.60	
	Trivial maximum level, mean (SD)	12.7 (17.1)		18.3 (16.7)		.07	
	Trivial highest score, mean (SD)	612 (1011.0)		956 (989.0)		.06	
	Fruit game maximum level, mean (SD)	1.67 (1.3)		2.15 (1.3)		.04	
	Fruit game highest score, mean (SD)	1174 (2258.0)		2020 (2451.0)		.06	
	Number of challenges completed, mean (SD)	1.42 (3.1)		2.95 (4.5)		.05	
	Island sections completed, mean (SD)	6.26 (10.5)		12.5 (12.4)		.006	
	Number of times they consulted the information section, mean (SD)	3.61 (4.5)		6.44 (7.8)		.04	

### Protocol Analysis of App Efficacy

Observing that 34.1% (97/284) of the IG did not use the app, we evaluated app usage using a protocol analysis. When we selected the IG participants who had at least one activity record stored in the server and compared them with the CG participants, we did not find significant differences between the groups in terms of tobacco cessation at 3 months and 12 months (*P*=.26 and *P*=.94, respectively), as seen in Table 3.

**Table 3:** Adjusted association in smoking cessation at 3 months and 12 months between control group and app users.^a^

Clinical outcomes	Control^b^, n (%)	App users^c^, n (%)	ICC^d^	OR^e^ (95% CI)	*P* value
Abstinent at 3 months	101 (31.8)	72 (38.5)	0.012	1.31 (0.82-2.09)	.26
Abstinent at 12 months	60 (18.9)	39 (20.9)	0.019	1.02 (0.58-1.79)	.94

^a^Adjusted by clinic group, age, gender, body mass index, education, Fagerström test assessment, number of previous attempts to quit, smokers in the family, use of electronic cigarettes, and use of a pharmacological treatment for tobacco cessation.

^b^n=318.

^c^n=187.

^d^ICC: intracluster correlation coefficient.

^e^OR: odds ratio.

We repeated the same analysis with the participants of the IG that we considered good users, taking into account that the size of the sample was small (n=26). We observed that those belonging to this group had a higher probability of being abstinent at 12 months (OR 7.20, 95% CI 2.14-24.20; *P*=.001) than the participants of the CG, as seen in Table 4.

**Table 4:** Adjusted association in smoking cessation at 3 months and 12 months between control group and good users.^a^

Clinical outcomes	Control^b^, n (%)	Good users^c^, n (%)	ICC^d^	OR^e^ (95% CI)	*P* value
Abstinent at 3 months	101 (31.8)	21 (80.8)	0.000	9.88 (3.37-28.91)	<.001
Abstinent at 12 months	60 (18.9)	15 (57.7)	0.053	7.20 (2.14-24.20)	.001

^a^Adjusted by clinic group, age, gender, body mass index, education, Fagerström test assessment, number of previous attempts to quit, smokers in the family, use of electronic cigarettes, and use of a pharmacological treatment for tobacco cessation.

^b^n=318.

^c^n=26.

^d^ICC: intracluster correlation coefficient.

^e^OR: odds ratio.

## Discussion

### Principal Findings

Our intervention trial, based on the use of a mobile app for smoking cessation in people older than 18 years who were motivated to quit smoking, included 773 participants who were monitored during their abstinence period for up to 12 months. The study demonstrated success in quitting smoking at 3 and 12 months among regular mobile app users compared with participants in the CG. Our study contributes to the literature on the design and evaluation of mobile health apps designed to help patients in the process of tobacco cessation [8-10,17], since most of the current research is based on the use of text messages to mobile phones [18].

Previous studies have shown that mobile phone app–based interventions may be useful tools for lifestyle interventions, such as weight loss [19], increased physical activity [20], or long-term condition management [21]. Moreover, a recent meta-analysis that included 5 studies that assess the effectiveness of smoking cessation using mobile apps alone to compare lower-intensity smoking cessations support found no evidence of a favorable effect of mobile apps in comparison with other types of interventions [22]. This meta-analysis also included one study that compared mobile app plus text messaging with a web-based intervention, which found evidence of a benefit of the app plus text messaging intervention [22]. Compared with the studies included in the meta-analysis, our study presents the longest follow-up (12 months) and the highest sample size.

We know that most attempts to quit smoking are not successful; according to the Centers for Disease Control and Prevention, only 12.2% of those who try to stop smoking remain abstinent [3]. For this reason, interventions that help young people and the general population stop smoking are needed. In our study, young people (aged 18 to 44 years) represent 57.7% (108/187) of those that used the app and 42.3% (11/26) of the good users. Mobile app usage seems more aligned with the lifestyle of the younger population, so this type of app has a double benefit in young people, since it reduces both smoking prevalence and comorbidity as patients grow older. Our mobile app includes some intervention components that have been successful in promoting smoking cessation, such as social compromise [23,24] and strategies to cope with abstinence syndrome and moments of craving [24,25].

Studies based on apps with this type of design should make sure that participants have a minimum level of digital skills with which to use the apps and that they are motivated to use them. As other researchers point out, it is important to study the usage patterns of the users in order to identify the features that are most helpful in the process of quitting smoking [26]. Researchers need to continue working on designs capable of identifying the key elements that help participants, as well as redesigning features (including new features) that can increase the efficacy of the app. Researchers should also consider how to personalize the intervention (and the available features within the app) to each of the population subgroups in order to maximize the engagement of the participants and, therefore, the probability of the success of the intervention. It is important to note that recent interventions that use an app for tobacco cessation are well received and viewed favorably by the participants [27], and it is vital that future apps take the needs of the users into account [28]. Mobile apps that use services hosted on online servers have an extra layer of complexity in complying with the technical and legal requirements of working with personal data.

In this initial Tobbstop study, users of the app appeared to exhibit patterns of participation and follow-up over time and demonstrated encouraging rates of tobacco cessation. Future research is warranted in order to evaluate the efficacy of Tobbstop in larger sample sizes.

We are currently working on adapting the app to other specific situations in which new features can improve its efficacy. In particular, we are designing a large-scale study of tobacco cessation among pregnant women. We have already performed a pilot study with 42 participants and have seen high success rates, even when the app was not designed for this specific group [29].

In our study, 34.1% (97/284) of the IG users never used the app, so we have to take this into account when making our analysis. This prevalence is similar to those obtained in previous studies [30]. Engagement and user retention are common and critical problems in mobile health. Previous studies have shown that more than two-thirds of people who downloaded a mobile phone app used it once and then stopped using it [31]. As a result, we have performed a sensitivity analysis between users and good users to obtain more accurate information about the efficacy of long-term usage of mobile phone apps in achieving smoking cessation. Our results have confirmed the hypothesis that long-term use of mobile apps improves the continuance of tobacco abstinence [32].

Despite the fact that the number of participant relapses were considered high, with 64.6% (183/284) of the IG and 73.3% (233/318) of the CG relapsing, our results have higher success rates than other studies that are also based on apps and have similar population samples [24,32]. In our study, we only enrolled participants with a high motivation to quit smoking. This parameter is unknown in other studies.

### Conclusions

A mobile app to help the process of quitting smoking presents higher success rates than standard interventions, indicating the viability of conducting a randomized community trial based on smartphone technologies.

## Appendix

Multimedia Appendix 1 Main features and information about the Tobbstop application.

Multimedia Appendix 2 CONSORT checklist.

## Abbreviations

CG: control group
CO: carbon monoxide
IG: intervention group
