# Supplementary material for: Evaluation of the Tobbstop Mobile App for Smoking Cessation: Cluster Randomized Controlled Clinical Trial
Source: JMIR Mhealth Uhealth. 2020 Jun 26;8(6):e15951. doi: 10.2196/15951 (PMC7381259; doi:10.2196/15951)

The main features included in the app are the following (Figure S1):

The main screen of the application consists of an island where the withdrawal process will take place. On D-Day, the participant lands on the beach of this island, which has a shape that resembles a lung and is painted with dark tones; it is also littered with trash and pollution. As days pass, the participant travels along a path on the island, cleaning the different parts and making it brighter and more colourful. The island is divided into six areas (landing, beach, swamp, city, forest and mountain), which are accessed according to different stages of the withdrawal guide (euphoria, grief, normalization, consolidation). On the last day of the path (90 days after the start) the player abandons the island in a balloon, leaving it behind fully clean and restored.

For each day participants log into the application they receive ‘virtual coins’ which they can use to buy decorations to personalize the visual aspect of their island. Those decorations have different levels of visual complexity and can be customized by changing the colour of the items.

**We can find different sections (see figure S1):**

1. Two **mini videogames**, one designed specifically to entertain (with a game we called Fruit, a game with similar mechanics to the Candy Crush® style of games) or to educate (with a Trivial Pursuit® style game). Both games come with several levels of difficulty to foster replay ability, and players are rewarded with coins to decorate the island based on their scores.
2. A section called ‘**activate**’ with challenges related to physical activity (i.e. walk X kilometers during Y days). Players can only have one of these challenges active at any moment.
3. A library where the participants can find information on tobacco, frequently asked questions, advice, definitions or tobacco-related images.
4. **Private chat** exclusive to the participants where they can share their experience, ask for help or give support to others. The members of the research team had access to the chat, but never participated in it.
5. A **visual registry** with the evolution of the health of the participant, where they can consult parameters such as time without smoking, money saved or the last registered anthropometric and control values (blood pressure, carbon monoxide (CO)-oximetry measure).
6. A place to **ask questions to an expert**, where participants can present their questions in a chat-like interface to the members of the research team and receive a response within a maximum of 48 hours.
7. A **trophy section**, with a showcase of all the challenges completed by the participant within the game. Those challenges are designed to cover all the features included in the game. Some of the trophies are achieved passively (for instance, rewards for spending a number of days without smoking) and some other require an action from the participant (obtaining a number of points on a mini game).
8. A **panic button** that participants can press if they experienced the temptation to smoke again. Upon pressing the button, participants receive advice and support messages to help calm their anxiety in the moment.

**Figure S1. Functionalities of the mobile app Tobbstop**


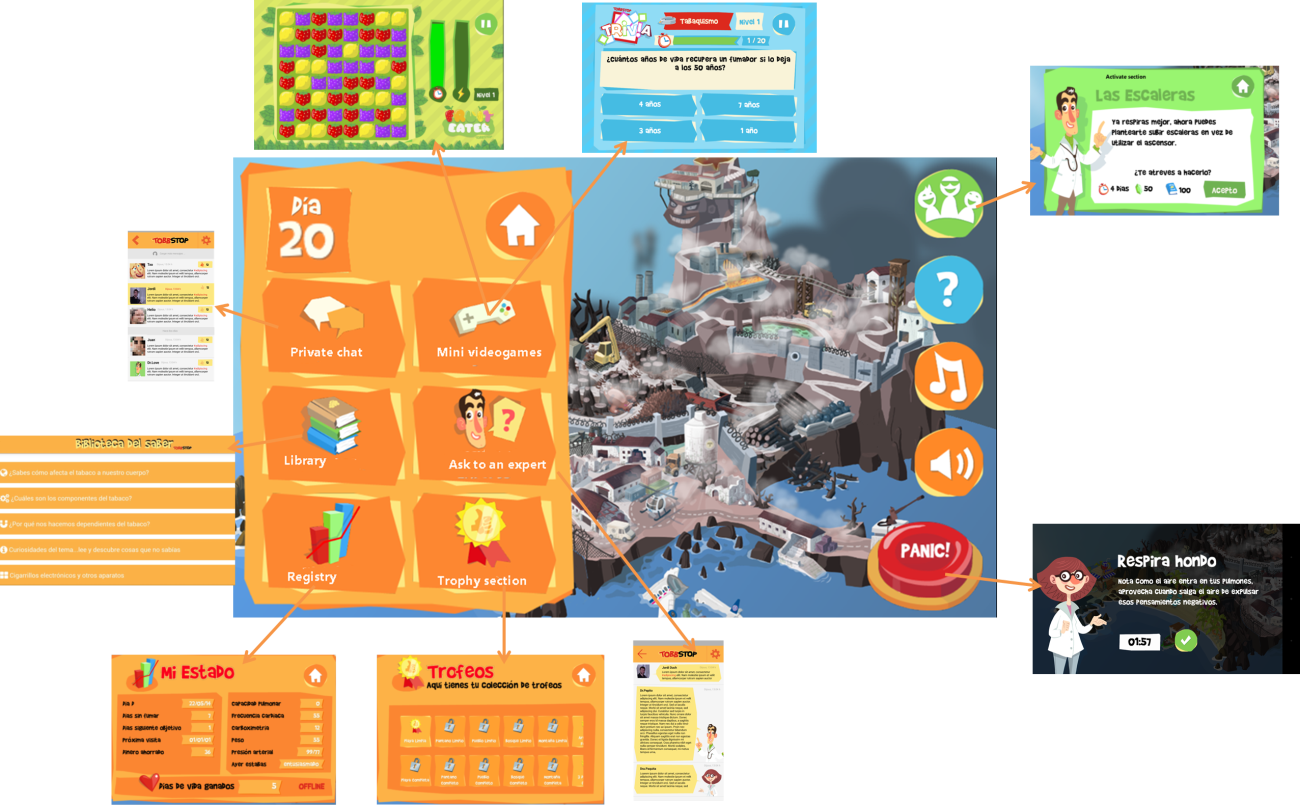

Supplement: Multimedia Appendix 1 [file mhealth_v8i6e15951_app1.docx]
